# Changing trends in the incidence, management and outcomes of coronary artery perforation over an 11-year period: single-centre experience

**DOI:** 10.1136/openhrt-2021-001916

**Published:** 2022-04-28

**Authors:** Hamza Umar, Harish Sharma, Mohammed Osheiba, Ashwin Roy, Peter F Ludman, Jonathan N Townend, M Adnan Nadir, Sagar N Doshi, Sudhakar George, Alex Zaphiriou, Sohail Q Khan

**Affiliations:** 1 College of Medical and Dental Sciences, University of Birmingham, Birmingham, UK; 2 Cardiology Department, Queen Elizabeth Hospital Birmingham, Birmingham, UK; 3 Institute of Cardiovascular Sciences, University of Birmingham, Birmingham, UK

**Keywords:** Coronary Vessels, Coronary Artery Disease, Percutaneous Coronary Intervention

## Abstract

**Introduction:**

Coronary artery perforation (CP) is a rare but life-threatening complication of percutaneous coronary intervention (PCI). This study aimed to assess the incidence, management and outcomes of CP over time.

**Methods:**

A single-centre retrospective cohort study of all PCIs performed between January 2010 and December 2020. Patients with CP were divided into two cohorts (A+B), representing the two halves of the 11-year study.

**Results:**

The incidence of CP was 68 of 9701 (0.7%), with an increasing trend over the two 5.5-year periods studied (24 of 4661 (0.5%) vs 44 of 5040 (0.9%); p=0.035). Factors associated with CP included chronic total occlusions (CTOs) (16 of 68 (24%) vs 993 of 9633 (10%); p<0.001), type C lesions (44 of 68 (65%) vs 4280 of 9633 (44%); p<0.001), use of intravascular ultrasound (IVUS) (12 of 68 (18%) vs 541 of 9633 (6%); p<0.001), cutting balloon angioplasty (3 of 68 (4%) vs 98 of 9633 (1%); p<0.001) and hydrophilic wires (24 of 68 (35%) vs 1454 of 9633 (15%); p<0.001). Cohorts A and B were well matched with respect to age (69±11 vs 70±12 years; p=0.843), sex (males: 13 of 24 (54%) vs 31 of 44 (70%); p=0.179) and renal function (chronic kidney disease: 1 of 24 (4%) vs 4 of 44 (9%); p=0.457). In cohort A, CP was most frequently caused by post-dilatation with non-compliant balloons (10 of 24 (42%); p=0.009); whereas in cohort B, common causes included guidewire exits (23 of 44 (52%)), followed by stent implantation (10 of 44 (23%)). The most common treatment modality in cohorts A and B was balloon inflation, which accounted for 16 of 24 (67%) and 13 of 44 (30%), respectively. The use of covered stents (16%) and coronary coils (18%) during cohort B study period did not impact all-cause mortality, which occurred in 2 of 24 (8%) and 7 of 44 (16%) (p=0.378) in cohorts A and B, respectively.

**Conclusion:**

The incidence of CP is increasing as more complex PCI is performed. Factors associated with perforation include CTO or type C lesions and use of IVUS, cutting balloon angioplasty or hydrophilic wires.

**Key questions**

**What is already known about this subject?**

- Coronary artery perforation (CP) is a rare complication of percutaneous coronary intervention (PCI).
- Earlier reports have established several risk factors predictive of CP, which are typically split into patient, angiographic and procedural factors.

**What does this study add?**

- This work demonstrates an increasing incidence of CP likely explained by a growing complexity of PCI procedures performed.
- The perforations were also graded according to the Modified Ellis criteria.

**How might this impact on clinical practice?**

- These results will inform clinicians on the factors that contribute to an increased risk of CP.
- Furthermore, awareness of this complication can enable prompt recognition and treatment of such patients, ultimately improving patient outcome.

## Introduction

Coronary perforation (CP) is the iatrogenic extravasation of blood or contrast from a coronary vessel, following a percutaneous coronary diagnostic or interventional procedure. It is associated with a 13-fold increase of in-hospital major adverse events and a 5-fold increase in 30-day mortality.^1^

Several risk factors for CP have been identified. Clinical predictors include advancing age, female sex and presence of renal impairment. Angiographic factors include complex vessel anatomy, calcification, the presence of type C lesions (angulated or tortuous vessels) and attempt at percutaneous coronary intervention (PCI) for chronic total occlusion (CTO).^1–4^ Operator factors include the decision to use glycoprotein IIb/IIIa (GpIIb/IIIa) inhibitors, oversized stents, hydrophilic or stiff wires, and the use of athero-ablative devices such as rotational atherectomy, laser atherectomy and cutting balloon angioplasty.^4–7^

Modern coronary angioplasty increasingly involves the use of adjunctive PCI techniques to treat complex lesions in an ageing population. Two large UK registry studies have reported a CP incidence of 0.33%–0.56%.^1 8^ Furthermore, a large meta-analysis of 65 studies demonstrated that the risk of CP rises to approximately 2.9% during CTO intervention.^9^

This study aimed to compared the incidence, management and outcomes of CP across two halves of the 11-year study period (A+B). Additionally, we compare the changing trends in PCI complexity by looking at surrogate markers, namely intravascular ultrasound (IVUS), hydrophilic wires, rotablation, cutting balloon angioplasty and CTO procedures.

## Methods

### Study population

This retrospective cohort study identified all PCI procedures performed between 1 January 2010 and 31 December 2020 inclusively, at the Queen Elizabeth Hospital, Birmingham, UK from a prospectively maintained electronic database. All patients who had a PCI procedure complicated with CP within this study period were retrospectively identified. The total CP population was then split into two further cohorts, A and B, for statistical analysis and comparison. The cohorts represented the first and second 5.5-year period within the study period. Cohort A included all perforations identified between 1 January 2010 and 2 July 2015, and cohort B referred to those that occurred between 3 July 2015 and 31 December 2020.

### Data collection

Two independent researchers collected data on the following parameters: patient demographics, comorbidities, angiographic characteristics, operator factors, treatment modalities, outcomes (cardiac tamponade, death and emergency coronary artery bypass graft). Cardiac tamponade was defined as the accumulation of extravasated fluid in the intrapericardial space.^10^ Treatment of patients was dependent on several factors: presence of tamponade, haemodynamic instability and perforation-specific factors such as severity, location and grade of perforation. The use of adjunctive devices, coronary wires and coronary complexity based on the American College of Cardiology/American Heart Association Criteria was also assessed.^11^

The two independent researchers analysed the angiographic appearance of the patients in search of results consistent with perforation and to analyse the vessel anatomy. CP was further stratified according to the Modified Ellis criteria, which differentiated CP into five distinct types (online supplemental table 1).^12 13^

10.1136/openhrt-2021-001916.supp1 Supplementary data

### Statistical analysis

Statistical analysis was conducted using SPSS software V.23.0 (IBM, Armonk, NY, USA). The normality and distribution of continuous data were tested using a Shapiro-Wilk test. For normally distributed data, a mean and SD were calculated. Categorical data were summarised as a percentage and statistically tested using a Χ^2^ test. Sample means were tested via a two-tailed t-test. All p<0.05 were deemed statistically significant.

## Results

### Incidence

**Figure 1:**
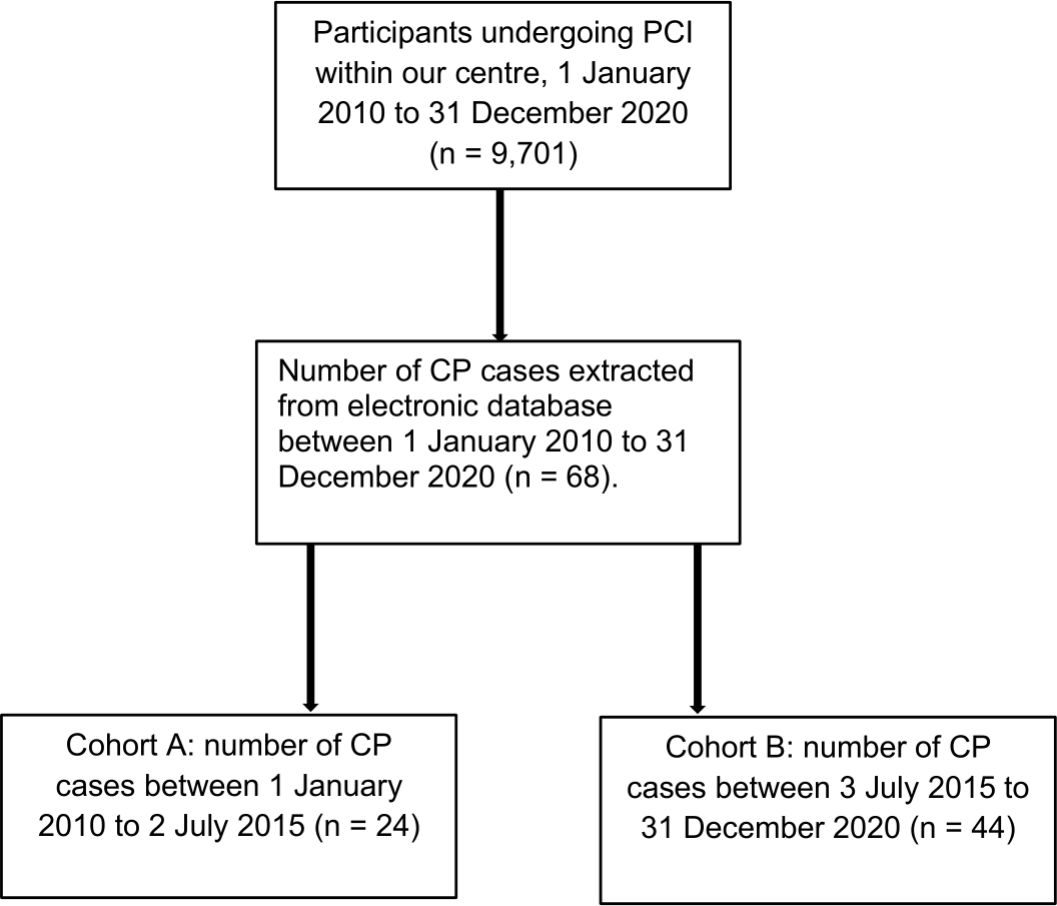
Schematic representation of study. CP, coronary perforation; PCI, percutaneous coronary intervention.

**Figure 2:**
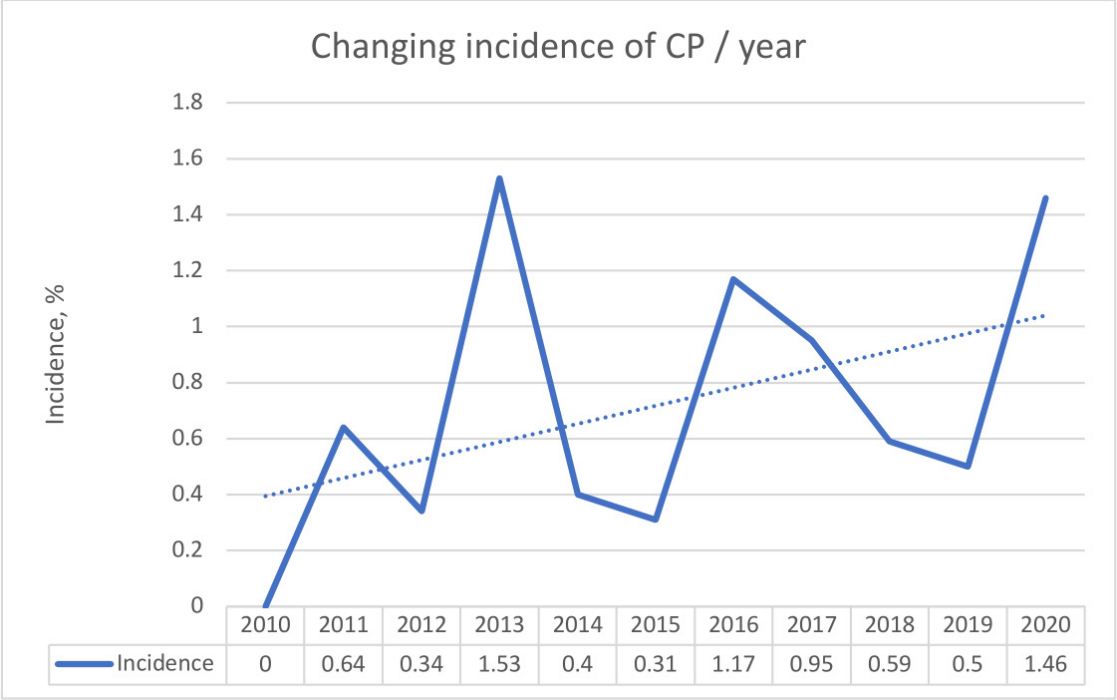
Changing incidence of CP across the study period. CP, coronary perforation.

CP occurred in 68 cases out of 9701 PCI procedures, with an overall incidence of 0.7% (figure 1). Cohort A demonstrated a significantly lower incidence of CP compared with cohort B (24 of 4661 (0.5%) vs 44 of 5040 (0.9%); p=0.035). Furthermore, the results demonstrate a non-linear, upward trend in the overall incidence of CP during the study period, with notable peaks in 2013, 2016 and 2020 (figure 2).

Baseline characteristics are shown in table 1. In comparison with patients without CP in the overall cohort, those who experienced CP were older (70±11 vs 65±12 years; p<0.001) with a higher proportion of patients with a history of smoking (24 of 68 (35%) vs 2229 of 9633 (23%); p=0.018) and peripheral vascular disease (PVD) (8 of 68 (12%) vs 463 of 9633 (5%); p=0.008). Subgroup analysis was performed by dividing the CP cohort by date of procedure, producing cohort A (1 January 2010–2 July 2015) and cohort B (3 July 2015–31 December 2020). In cohort A, female sex was associated with a higher likelihood of CP (11 of 24 (46%) vs 1201 of 4637 (26%); p=0.026). There were otherwise no significant differences between CP and non-CP groups in cohort A. In cohort B, CP was associated with older age, chronic kidney disease (4 of 44 (9%) vs 164 of 4996 (3%); p=0.034), PVD (7 of 44 (16%) vs 245 of 4996 (5%); p<0.001) and current smokers (19 of 44 (43%) vs 1072 of 4996 (21%); p<0.001).

**Table 1:** A comparison of baseline characteristics between overall cohort, cohort A (1 January 2010–2 July 2015) and cohort B (3 July 2015–31 December 2020)

Variable	Overall			Cohort A			Cohort B		
	Non-CP (n=9633)	CP (n=68)	P value	Non-CP (n=4637)	CP (n=24)	P value	Non-CP (n=4996)	CP (n=44)	P value
Mean age and SD	65±12	70±11	0.001	65±13	69±11	0.133	65±12	70±13	0.006
Gender									
Male, *n (%*)	7029 (73)	44 (65)	0.127	3436 (74)	13 (54)	0.026	3590 (72)	31 (70)	0.837
Smoking status									
Current, *n (%*)	2229 (23)	24 (35)	0.018	1157 (25)	5 (21)	0.642	1072 (21)	19 (43)	<0.001
Medical history									
Hypertension, *n (%*)	5719 (59)	46 (68)	0.166	2652 (57)	17 (71)	0.178	3067 (61)	29 (66)	0.540
Diabetes, *n (%*)	2610 (27)	15 (22)	0.352	1017 (22)	3 (13)	0.265	1593 (32)	12 (27)	0.513
ACS, *n (%*)	6599 (69)	49 (72)	0.529	3334 (72)	20 (83)	0.213	3265 (65)	29 (66)	0.938
Stable angina, *n (%*)	3034 (31)	19 (28)	0.529	1327 (29)	4 (17)	0.196	1707 (34)	15 (34)	0.992
CKD, *n (%*)	341 (4)	5 (7)	0.091	177 (4)	1 (4)	0.929	164 (3)	4 (9)	0.034
Previous MI, *n (%*)	2833 (29)	22 (32)	0.596	1266 (27)	7 (29)	0.838	1567 (31)	15 (34)	0.698
Hypercholesterolaemia, *n (%*)	4763 (49)	36 (53)	0.566	2421 (52)	17 (71)	0.068	2342 (47)	19 (43)	0.625
PVD, *n (%*)	463 (5)	8 (12)	0.008	218 (5)	1 (4)	0.902	245 (5)	7 (16)	<0.001
History of CABG, *n (%*)	874 (9)	8 (12)	0.442	424 (9)	3 (13)	0.570	450 (9)	5 (11)	0.587
Family history of CAD, *n (%*)	4014 (42)	26 (38)	0.567	1710 (37)	9 (38)	0.950	2304 (46)	17 (39)	0.322

ACS, acute coronary syndrome; CABG, coronary artery bypass graft; CAD, coronary artery disease; CKD, chronic kidney disease; CP, coronary perforation; MI, myocardial infarction; PVD, peripheral vascular disease.

Over the 11-year period studied, amongst patients with CP, there was a significantly higher number of type B2 lesions treated in cohort A (7 of 24 (29%) vs 3 of 44 (7%); p=0.013) and in-stent restenoses treated in cohort B (0 of 24 (0%) vs 9 of 44 (18%); p=0.026) (table 2). There were otherwise no significant differences between the cohorts with respect to use of IVUS, rotablation, cutting balloon angioplasty, hydrophilic wires, multivessel stenting or GpIIb/IIIa inhibitor. Furthermore, there was no significant difference in the types of CP observed, or the target lesion involved across the 11-year period.

**Table 2:** Comparison of angiographic and procedural characteristics between cohort A (1 January 2010–2 July 2015) and cohort B (3 July 2015–31 December 2020)

Variable	Cohort A (n=24)	Cohort B (n=44)	P value
Lesion complexity (ACC/AHA)			
Type A, *n (%*)	0	0	N/A
Type B1, *n (%*)	3 (13)	11 (25)	0.223
Type B2, *n (%*)	7 (29)	3 (7)	0.013
Type C, *n (%*)	14 (58)	30 (68)	0.417
CTO attempted, *n (%*)	4 (17)	12 (27)	0.324
In-stent restenoses, *n (%*)	0	8 (18)	0.026
IVUS, *n (%*)	4 (17)	8 (18)	0.876
Rotablation, *n (%*)	1 (4)	4 (9)	0.457
Cutting balloon angioplasty, *n (%*)	1 (4)	2 (5)	0.942
Hydrophilic wires, *n (%*)	9 (38)	15 (34)	0.779
Multivessel stenting, *n (%*)	5 (21)	13 (30)	0.436
GpIIb/IIIa inhibitors, *n (%*)	7 (29)	7 (16)	0.196
Type of coronary perforation			
Type I, *n (%*)	1 (4)	2 (5)	0.942
Type II, *n (%*)	4 (17)	5 (11)	0.537
Type III, *n (%*)	11 (46)	19 (43)	0.833
Type IV, *n (%*)	0 (0)	0 (0)	N/A
Type V, *n (%*)	8 (33)	18 (41)	0.539
Target lesion			
LAD, *n (%*)	8 (33)	18 (41)	0.539
RCA, *n (%*)	9 (38)	9 (20)	0.128
Cx, *n (%*)	3 (13)	4 (9)	0.658
Diagonal, *n (%*)	1 (4)	8 (18)	0.103
Septal, *n (%*)	1 (4)	2 (5)	0.942
Intermediate, *n (%*)	0	2 (5)	N/A
LMS, *n (%*)	1 (4)	1 (2)	0.659
SVG, *n (%*)	1 (4)	0	N/A

ACC/AHA, American College of Cardiology/American Heart Association; CTO, chronic total occlusion; Cx, circumflex artery; GpIIb/IIIa, glycoprotein IIb/IIIa; IVUS, intravascular ultrasound; LAD, left anterior descending; LMS, left main stem; N/A, not applicable; RCA, right coronary artery; SVG, saphenous vein graft.

Patients with CP were more likely to have had PCI attempted for CTOs (16 of 68 (24%) vs 993 of 9633 (10%); p<0.001) and type C lesions (44 of 68 (65%) vs 4280 of 9633 (44%); p<0.001) (online supplemental table 2). Procedures with CP also more frequently involved the use of IVUS (12 of 68 (18%) vs 541 of 9633 (6%); p<0.001), cutting balloon angioplasty (3 of 68 (4%) vs 98 of 9633 (1%); p<0.001) and hydrophilic wires (24 of 68 (35%) vs 1454 of 9633 (15%); p<0.001).

In cohort A, the most common cause of CP was post-dilatation with non-compliant balloon (10 of 24 (42%); p=0.009) (online supplemental table 3). Overall, balloon inflations accounted for 11 of 24 (46%) cases of CP, followed by guidewire perforations (8 of 24 (33%)) and stent implantation (5 of 24 (21%)). By contrast, in cohort B, the most common cause of CP was guidewire exits (23 of 44 (52%)), followed by stent implantation (10 of 44 (23%)), balloon inflation (9 of 44 (20%)) and use of cutting balloon (2 of 44 (5%)).

### Management of perforations and outcomes

**Figure 3:**
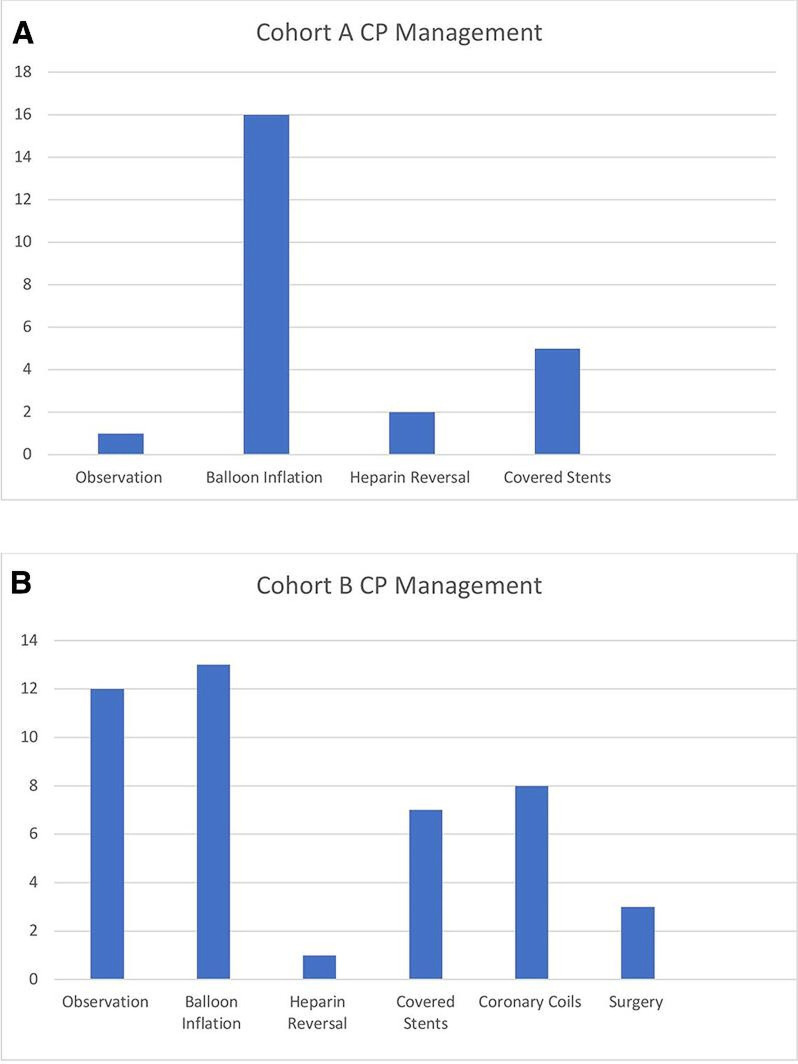
(A)Graphical representation of cohort A CP management. (B) Graphical representation of cohort B CP management. CP, coronary perforation.

Figure 3A, B offers a graphical comparison of the different management approaches adopted in the first and second 5.5-year periods. In cohort A, all type I and II (5 of 24) perforations were treated using balloon inflation (table 3). The management of type III perforations (11 of 24) was split between proximal balloon inflation (6 of 11 (55%)) and covered stent deployment (5 of 11 (45%)). Type V perforations (8 of 24) were predominantly managed with balloon inflation (5 of 8 (62.5%)); however, heparin reversal and conservative observation was also used (3 of 8 (37.5%)).

**Table 3:** Definitive management and outcomes in cohort A (1 January 2010–2 July 2015) and cohort B (3 July 2015–31 December 2020)

Treatment	Cohort A (n=24)				Cohort B (n=44)			
	Type I (n=1)	Type II (n=4)	Type III (n=11)	Type V (n=8)	Type I (n=2)	Type II (n=5)	Type III (n=19)	Type V (n=18)
Observation, *n (%*)	0	0	0	1 (13)	2 (100)	3 (60)	2 (11)	5 (28)
Balloon inflation, *n (%*)	1 (100)	4 (100)	6 (55)	5 (63)	0	2 (40)	6 (32)	5 (28)
Heparin reversal, *n (%*)	0	0	0	2 (25)	0	0	0	1 (6)
Covered stent, *n (%*)	0	0	5 (45)	0	0	0	7 (37)	0
Coronary coils, *n (%*)	0	0	0	0	0	0	1 (5)	7 (39)
Surgery, *n (%*)	0	0	0	0	0	0	3 (16)	0
**Outcome**								
Tamponade, *n (%*)	0 (0)	0 (0)	1 (9)	1 (13)	0 (0)	0 (0)	7 (37)	5 (28)
Autotransfusion, *n (%*)	0 (0)	0 (0)	1 (100)	0 (0)	0 (0)	0 (0)	0 (0)	0 (0)
Death, *n (%*)	0 (0)	1 (25)	1 (9)	0 (0)	0 (0)	0 (0)	7 (37)	0 (0)
CABG/sternotomy, *n (%*)	0 (0)	0 (0)	0 (0)	0 (0)	0 (0)	0 (0)	3 (16)	0 (0)
**Composite outcomes**								
Tamponade OR death OR CABG/sternotomy, *n (%*)	0 (0)	1 (25)	1 (9)	1 (13)	0 (0)	0 (0)	9 (47)	5 (28)

CABG, coronary artery bypass graft.

In cohort B, the majority of type I and II (7 of 44) perforations were managed conservatively through observation (5 of 7 (71%)), while balloon inflation was used in a minority of cases (2 of 7 (29%)). Similarly, type III perforations (19 of 44) were largely treated by covered stent deployment (7 of 19 (37%)) or balloon inflation (6 of 19 (32%)). Emergency cardiac surgery was performed to treat type III perforations in a minority of cases (3 of 19 (16%)). In contrast to cohort A, the use of coronary coils was the main modality in treating type V perforations in this cohort (7 of 18 (39%)).

Overall, amonngst CP cases, compared to cohort A, cohort B were more likely to reach the composite outcome of tamponade, death or requirement for cardiothoracic surgery (3 of 24 (13%) vs 14 of 44 (32%); p=0.079). Type III perforations wereassociated with the highest frequency of composite adverse outcomes compared with any other perforation type (10 of 17 (59%)). Of all reported deaths, eight of nine (89%) were due to type III left anterior descending perforations. One death occurred in a type II perforation after cardiogenic shock. Cardiac tamponade was a poor prognostic factor, contributing to 50% and 71% of deaths within cohorts A and B, respectively.

## Discussion

This retrospective cohort study investigated the incidence, management and outcomes of an 11-year dataset of CP at a large regional cardiac centre. The study demonstrated that the incidence of CP was rising across the 11-year study period. The study also confirms the poor outcomes following type III perforations and found that the presence of cardiac tamponade was a poor prognostic indicator. Furthermore, a greater proportion of poorer composite outcomes were observed during the latter 5.5-year study period. Surrogate markers of PCI complexity were factors associated with perforation.

During the period studied (2010–2020 inclusively), the incidence of CP rose from 0% to 1.46% per annum. The overall incidence of CP is slightly higher than those reported in large UK registries from 2008 and 2013, but similar to a Netherlands-based study from 2016.^1 4 8^ If the same trends are observed in other centres, CP may be an increasingly encountered complication, requiring more pre-procedural risk stratification. The significant rise in incidence likely reflects the increasing procedural complexity and burden of disease being treated percutaneously. In total, 80% of CPs occurred in patients with type B2/C lesions, and the use of IVUS, hydrophilic wires, rotablation, cutting balloon angioplasty and CTO procedures was all associated with CP.

Furthermore, the study identified notable peaks in the incidence of CP in 2013, 2016 and 2020. While the peaks in 2013 and 2016 could be explained by the introduction of new consultants in the department, the peak in 2020 is likely related to the COVID-19 pandemic. Due to the high intensive care bed occupancy, there was an overall decline in the number of interventional procedures performed but an increase in PCI performed in patients who would have otherwise been candidates for cardiac surgery.

This study found that patient factors associated with CP include increasing age and smoking history, which are both associated with more significant coronary artery disease. Previous studies have also identified female sex and comorbidities as risk factors for CP.^1 14^ In the overall cohort, we found no significant differences between sex or other major comorbidities except PVD, which may be a surrogate for extensive atherosclerotic disease. Our study, however, did not evaluate the cumulative effect of multiple comorbidities; thus, further work is required to establish risk prediction models based on the multiple factors involved. A comparison between cohort A and cohort B was conducted to evaluate differing CP trends across the study period. Within cohort B, there were significantly more patients who had in-stent restenoses after previously receiving percutaneous treatment; this finding may explain the greater number of coronary perforations during the latter half of the study period.

Over the 11-year period studied, there were poorer outcomes observed in the latter 5.5 years (cohort B), despite the introduction to our centre of covered stents and coronary coils in 2016.

The study also confirms the gravity of type III perforations as the incidence and overall mortality of this subtype were 0.31% and 27%, respectively, both of which were greater when compared with the results published by Al-Lamee *et al*.^2^ Cardiac tamponade was particularly associated with a high risk of mortality (43%), in line with previously published data.^1^ With a trend towards more complex PCI, cardiac tamponade is likely to be more frequently encountered. The use of autologous blood transfusions in patients with tamponade may reduce adverse outcomes by helping to stabilise the patient and lowering the risk of allogenic blood reactions.^15^ In our cohort, autotransfusion was initiated in only one patient who suffered from tamponade; thus, further research in this area is required.

## Limitations

This study is a retrospective data analysis from a single regional cardiac centre, thus the findings are subject to the inherent limitations of a retrospective cohort study. There was no control group or angiographic follow-up; thus, it is difficult to comment on target vessel failure or rates of re-intervention following covered stent deployment. In addition, the study did not analyse the effect of individual operators on the incidence of coronary perforation and patient outcomes. Despite a high volume of procedures performed at this centre, the number of cases within the CP cohort and its subsequent subgroups was low. This reflects the rarity of this complication. A multivariate analysis was performed, but not shown, due to a relatively small sample size yielding statistical insignificance. Thus, the study was unable to identify independent predictors of CP. Data on coronary vessel calcification were unavailable thus the study was unable to determine the full extent of vessel disease on the incidence of CP. Furthermore, an analysis of multiple comorbidities on the risk of CP was not conducted.

## Data Availability

Data are available upon reasonable request.
