# Supplementary material for: Changing trends in the incidence, management and outcomes of coronary artery perforation over an 11-year period: single-centre experience
Source: Open Heart. 2022 Apr 28;9(1):e001916. doi: 10.1136/openhrt-2021-001916 (PMC9052042; doi:10.1136/openhrt-2021-001916)
Supplement: Supplementary data [file openhrt-2021-001916supp001.pdf]

Supplementary Material

Supplementary Table 1: The Modified Ellis Criteria

**Type**	**Description**
I	The development of an extraluminal crater without brisk extravasation.
II	Pericardial or myocardial blushing without brisk extravasation.
III	Breach in the arterial wall that results in an extravasation jet through a frank perforation (width of >=1mm).
IV	Accumulation of blood into another anatomical cavity.
V	Synonymous with a distal perforation.

Supplementary Table 2: Comparison of angiographic and procedural characteristics between No CP and CP

**Variable**	**No CP (n = 9633)**	**CP (n = 68)**	***P*-value**
Lesion complexity (ACC/AHA)			
Type A, *n (%)*	331 (3)	0	N/A
Type B1, *n (%)*	1619 (17)	14 (21)	0.406
Type B2, *n (%)*	2198 (23)	10 (15)	0.112
Type C, *n (%)*	4280 (44)	44 (65)	<0.001
***CTO attempted**, n (%)*	993 (10)	16 (24)	<0.001
***In-stent Restenoses**, n (%)*	918 (10)	8 (12)	0.532
***IVUS**, n (%)*	541 (6)	12 (18)	<0.001
***Rotablation**, n (%)*	363 (4)	5 (7)	0.123
***Cutting Balloon Angioplasty**, n (%)*	98 (1)	3 (4)	<0.001
***Hydrophilic wires**, n (%)*	1454 (15)	24 (35)	<0.001
***Multivessel stenting**, n (%)*	3488 (36)	18 (26)	0.096
***GPIIb/IIIa inhibitors**, n (%)*	2875 (30)	14 (21)	0.096

ACC/AHA, American College of Cardiology/ American Heart Association; CTO, chronic total occlusion; IVUS, intravascular ultrasound

Supplementary Table 3: **Mechanisms of Perforation for Cohort A (1 January 2010 - 2 July 2015) and Cohort B (3 July 2015 and the 31 December 2020)**

**Perforation cause**	**Cohort A,** (n = 24)	**Cohort B,** (n = 44)	***P*-value**
Guidewire			
Hydrophilic wire*, n (%)*	4 (17)	13 (30)	0.241
Work Horse wire*, n (%)*	4 (17)	10 (23)	0.555
Cutting balloon*, n (%)*	0	2 (5)	NA
Coronary stent*, n (%)*	5 (21)	10 (23)	0.857
Balloon inflation			
Pre-dilatation with NC balloon*, n (%)*	1 (4)	2 (5)	0.942
Post-dilatation with NC balloon, *n (%)*	10 (42)	6 (14)	0.009
Pre-dilation with SC balloon*, n (%)*	0	1 (2)	NA
Post-dilation with SC balloon, *n (%)*	0	0	NA

NC, Non-Compliant; SC, Semi-Compliant
